# Draft Genome Sequence of Pseudomonas mandelii Strain 29, Isolated from the Desert Truffle Terfezia claveryi

**DOI:** 10.1128/mra.00648-22

**Published:** 2022-09-26

**Authors:** Ángel Luigi Guarnizo, Asunción Morte, Alfonso Navarro-Ródenas

**Affiliations:** a Departamento Biología Vegetal, Facultad de Biología, CEIR Campus Mare Nostrum (CMN), Universidad de Murcia, Murcia, Spain; University of Arizona

## Abstract

Here, we report the genome sequence of the mycorrhizal helper bacterium (MHB) Pseudomonas mandelii strain 29. This is the genome of an MHB associated with ascocarps of the desert truffle Terfezia claveryi. The genome is complete and consists of 6,302,122 bp and 5,812 predicted protein-coding sequences.

## ANNOUNCEMENT

The genus Pseudomonas is composed of Gram-negative bacteria of the family *Pseudomonadaceae* (1). Pseudomonas bacteria are rod-shaped, mobile with polar flagella, and widely distributed in nature. Pseudomonas mandelii strain 29 (phosphorus solubilizing) was isolated from the peridium of ascocarps of the ectendomycorrhizal fungus Terfezia claveryi Chatin, and it is characterized by its ability to increase mycorrhizal colonization of Helianthemum almeriense host plants under nursery conditions (2). Since mycorrhizal helper bacteria (MHB) appear to have specificity for certain strains of mycorrhizal fungi (3), understanding the mechanisms of action of MHB could be useful to employ *P. mandelii* as a promising option for sustainable use in agriculture (2, 4).

A single-colony culture of *P. mandelii* was subcultured in nutrient broth and was grown overnight at 30°C in a shaking incubator at 200 rpm. Genomic DNA was extracted using a QIAamp DNA extraction kit (Qiagen) following the manufacturer’s instructions. Library construction was performed according to the reference guide of Illumina Nextera DNA Flex Library Prep (currently named Illumina DNA Prep). The draft genome sequence data of *P. mandelii* 29 were obtained on an Illumina MiSeq platform with a read length of 300 bp (paired-end 150), using a MiSeq v3 reagent kit (600 cycles). A total of 3,705,263 paired reads and 1,089,807,791 bp (~193× coverage) were generated for *P. mandelii* strain 29. Default parameters were used for all software unless otherwise specified. Quality control was assessed using FastQC v.0.119 (5). The reads were filtered using Trimmomatic v0.36 (6) and were assembled in PATRIC V3.6.12 (7), using the SPAdes v3.12.0 program (8) after filtering reads by length (≥300 bp). Quality assessment of the assemblies was performed using QUAST version v5.0.2 (9), SAMtools version 1.3 (10), and Pilon version 1.23 (11).

The draft genome sequence consists of 6.3 Mb, with a G+C content of 58.8%, and reads were annotated into 87 contigs with an *N*_50_ contig length of 239,038 bp. The online RAST gene annotation pipeline (12) predicted 5,812 predicted genes. The annotation includes 3,739 proteins with functional assignments, 2,073 hypothetical proteins, 3 rRNAs, and 72 tRNA loci. The reference genome used for functional annotation with PATRIC was *P. mandelii* YF10-2(1) 51 (BioProject accession number PRJNA46438). Annotation by RAST reported 372 subsystems in the genome. The phosphorus and nitrogen metabolism accounted for 44 and 13 subsystem feature counts, respectively. Amino acids and derivatives (501), carbohydrates (272), and cofactors, vitamins, prosthetic groups, and pigments (218) were the subsystems with the most feature counts. The genome also was annotated with GAP v5.3, the NCBI Prokaryotic Genome Annotation Pipeline (13, 14).

The availability of the genome sequence of *P. mandelii* strain 29 should provide valuable information to elucidate the understanding of MHB mechanisms.

### Data availability.

The raw data (Illumina MiSeq) generated during this experiment have been deposited in the NCBI SRA database under the BioProject accession number PRJNA853178. The assembled genome sequence was submitted to the NCBI GenBank database under the accession number JAMYDU000000000 and SRA accession number SRP395192.
